# Detection of COPD in the SUMMIT Study lung cancer screening cohort using symptoms and spirometry

**DOI:** 10.1183/13993003.00795-2022

**Published:** 2022-12-08

**Authors:** Sophie Tisi, Jennifer L. Dickson, Carolyn Horst, Samantha L. Quaife, Helen Hall, Priyam Verghese, Kylie Gyertson, Vicky Bowyer, Claire Levermore, Anne-Marie Mullin, Jonathan Teague, Laura Farrelly, Arjun Nair, Anand Devaraj, Allan Hackshaw, the SUMMIT Consortium, John R. Hurst, Sam M. Janes

**Affiliations:** 1 Lungs for Living Research Centre, UCL Respiratory, University College London, London, UK; 2 Centre for Prevention, Detection and Diagnosis, Wolfson Institute of Population Health, Barts and The London School of Medicine and Dentistry, Queen Mary University of London, London, UK; 3 University College London Hospitals NHS Foundation Trust, London, UK; 4 Cancer Research UK and UCL Cancer Trials Centre, University College London, London, UK; 5 Royal Brompton and Harefield NHS Foundation Trust, London, UK; 6 Full details of the SUMMIT Consortium and affiliations are included in the supplementary material

## Abstract

**Background:**

COPD is a major comorbidity in lung cancer screening (LCS) cohorts, with a high prevalence of undiagnosed COPD. Combining symptom assessment with spirometry in this setting may enable earlier diagnosis of clinically significant COPD and facilitate increased understanding of lung cancer risk in COPD. In this study, we wished to understand the prevalence, severity, clinical phenotype and lung cancer risk of individuals with symptomatic undiagnosed COPD in a LCS cohort.

**Methods:**

16 010 current or former smokers aged 55–77 years attended a lung health check as part of the SUMMIT Study. A respiratory consultation and spirometry were performed alongside LCS eligibility assessment. Those with symptoms, no previous COPD diagnosis and airflow obstruction were labelled as undiagnosed COPD. Baseline low-dose computed tomography (LDCT) was performed in those at high risk of lung cancer (PLCO_m2012_ score ≥1.3% and/or meeting USPSTF 2013 criteria).

**Results:**

Nearly one in five (19.7%) met criteria for undiagnosed COPD. Compared with those previously diagnosed, those undiagnosed were more likely to be male (59.1% *versus* 53.2%; p<0.001), currently smoking (54.9% *versus* 47.6%; p<0.001) and from an ethnic minority group (p<0.001). Undiagnosed COPD was associated with less forced expiratory volume in 1 s impairment (Global Initiative for Chronic Obstructive Lung Disease (GOLD) grades 1 and 2: 85.3% *versus* 68.4%; p<0.001) and lower symptom/exacerbation burden (GOLD A and B groups: 95.6% *versus* 77.9%; p<0.001) than those with known COPD. Multivariate analysis demonstrated that airflow obstruction was an independent risk factor for lung cancer risk on baseline LDCT (adjusted OR 2.74, 95% CI 1.73–4.34; p<0.001), with a high risk seen in those with undiagnosed COPD (adjusted OR 2.79, 95% CI 1.67–4.64; p<0.001).

**Conclusions:**

Targeted case-finding within LCS detects high rates of undiagnosed symptomatic COPD in those most at risk. Individuals with undiagnosed COPD are at high risk for lung cancer.

## Abstract 2

**Targeted case-finding using symptom assessment and spirometry within a large-scale lung cancer screening setting detects high rates of early-stage, clinically significant undiagnosed COPD. Those with undiagnosed COPD are at high risk for lung cancer** https://bit.ly/3bo5piE

## Introduction

There is a well-established link between COPD and lung cancer, with COPD a significant risk factor for lung cancer independent of smoking status [1–4], increasing the risk of lung cancer by 4–6-fold [5, 6].

COPD and lung cancer place significant burden on global healthcare infrastructure and expenditure, ranking as the third and fifth leading causes of worldwide mortality, respectively [7]. COPD underdiagnosis is well recognised and it is estimated that 71.2–86.5% of individuals with COPD have not been diagnosed [8–10]. Lung cancer's poor prognosis largely relates to diagnosis being delayed until disease is advanced. Similarly, a diagnosis of COPD may often be missed until functional reserve is limited or associated complications have manifested.

Lung cancer screening (LCS) with low-dose computed tomography (LDCT) scanning decreases the relative risk of lung cancer mortality in high-risk individuals by 20–26% [11, 12]. As the LCS population is also at high risk for COPD, LCS may offer an additional opportunity to “case-find” for COPD. The use of spirometry within this setting has demonstrated rates of undiagnosed airflow obstruction between 4% and 38% [13–18]. High levels of underdiagnosis may have additional implications as to the accuracy of existing lung cancer risk prediction models for entry into screening programmes, which often rely on self-reported COPD diagnoses as a marker of lung cancer risk [19, 20].

Understanding whether and how case-finding for COPD should be implemented in large-scale population-based LCS programmes remains unclear. Published rates of LCS-detected COPD have largely defined COPD by the presence of airflow obstruction alone. This combines those who would be regarded as having subclinical disease (asymptomatic) and those who have clinically significant disease (symptomatic). Whilst subclinical disease may be a marker of future COPD development and impart information about an individual's future risk of lung cancer, existing evidence shows no benefit from case-finding in this group [21] because of the absence of effective disease-modifying intervention, with the exception of smoking cessation, which is relevant irrespective of COPD status. Consequently, screening for COPD is only advocated in symptomatic individuals with exposure to relevant risk factors [21, 22]. An approach to COPD case-finding within LCS which combines symptom assessment with spirometry may therefore optimise greater health resource utilisation and facilitate more useful phenotyping beyond forced expiratory volume in 1 s (FEV_1_) classification, with potential for greater impact on downstream treatment outcomes and quality of life.

We report the SUMMIT Study's targeted approach combining respiratory symptom collection in conjunction with spirometry to identify undiagnosed symptomatic COPD in individuals who attended a lung health check (LHC) as part of eligibility assessment for LCS. The aims of this analysis were to understand the prevalence of undiagnosed symptomatic COPD in this cohort, whether the severity and clinical COPD phenotype varied between those with previously diagnosed and undiagnosed COPD, and whether the prevalence of undiagnosed COPD differed between those eligible and those ineligible for a LCS programme based on predicted lung cancer risk. Finally, we sought to understand lung cancer risk following a baseline round of LCS in those with undiagnosed COPD.

## Methods

### SUMMIT Study design

The SUMMIT Study is a large prospective observational cohort study designed to examine the performance of delivering a LDCT screening service to a high-risk population in London in the UK and to validate a multi-cancer early detection blood test [23]. Individuals aged 55–77 years registered with participating general practice surgeries and coded as being a smoker within the past 20 years were invited to attend a LHC at one of four LDCT scanning sites. The LHC included respiratory symptom assessment and spirometry, alongside formal assessment of lung cancer risk. The SUMMIT Study is registered at ClinicalTrials.gov with identifier number NCT03934866.

Two lung cancer risk models were used at the LHC to assess eligibility for LDCT screening as part of the SUMMIT Study. Individuals were considered eligible if they met either the 2013 US Preventive Services Task Force (USPSTF) criteria (at least 30 pack-year history and if a former smoker have not given up longer than 15 years) [24] or a Prostate, Lung, Colorectal and Ovarian Study Risk Prediction Model modified 2012 (PLCO_m2012_) [25] 6-year lung cancer risk score of ≥1.3%. A PLCO_m2012_ risk score of ≥1.3% was chosen as this has been demonstrated to maintain the same sensitivity as the USPSTF criteria [26]. Individuals who did not meet eligibility criteria were unable to participate in LDCT screening. Eligible individuals, if willing to participate, were consented to the study and offered a same day LDCT. Participants will return for two further annual visits. We report an interim analysis of all those who attended a LHC from the opening of recruitment in April 2019 to a temporary pause to recruitment in late March 2020 following the severe acute respiratory syndrome coronavirus 2 pandemic.

### Data collection through attendance at a LHC

A targeted approach to case-finding which combined respiratory symptom assessment and spirometry was used to identify individuals who may have undiagnosed COPD.

#### Spirometry

Quality-assured spirometry was performed in all individuals unless contraindicated. As spirometry was intended for identification of high-risk individuals rather than formal diagnosis, all manoeuvres were pre-bronchodilator. Attendees were encouraged to perform three manoeuvres guided by healthcare practitioners trained to the Association for Respiratory Technology and Physiology standards [27]. The highest value was recorded, with measurements and associated Global Lung Function Initiative reference values collected for FEV_1_, forced vital capacity (FVC) and FEV_1_/FVC values. A quality-assured approach to performing spirometry was completed in accordance with the American Thoracic Society/European Respiratory Society guidelines [28]. Equipment quality assurance criteria were ensured through the use of ISO 26782 compliant Vitalograph micro handheld spirometers (Vitalograph, Maids Moreton, UK), alongside daily calibration. Central review of spirometry quality was conducted for the first 1000 spirometry sessions.

#### Respiratory symptom and history assessment

A targeted consultation was undertaken by study-trained healthcare practitioners to screen for the presence of respiratory symptoms. Table 1 outlines the questions used to assess the presence and duration of cough, dyspnoea graded by the modified Medical Research Council (mMRC) scale, sputum production, and exacerbations in the past 12 months.

**TABLE 1:** Questions used to assess the presence and duration of cough, dyspnoea, sputum production and exacerbations in the past 12 months

**Question**	**Answer**
**Do you currently have a cough?**	Yes
	No
**(If yes) When did the cough start?**	Within 3 weeks
	3–6 weeks ago
	6 weeks to 6 months ago
	6 months to 12 months ago
	Greater than 12 months ago
**When you cough, do you usually cough up phlegm (sputum)?**	Yes No
**How many times have you had a chest infection or pneumonia in the past year for which you needed antibiotics and/or steroids?**	(number)
**Which of these best describes your breathing?**	Only breathless on strenuous exercise Breathless when hurrying on the flat or up a slight hill
	Slower than peers when walking, would need to stop after 15 min or 1 mile (1.6 km) at own pace
	Would need to stop due to breathlessness after 100 yards (91.4 m) on the flat
	Too breathless to leave house or when washing/dressing
	Unable to answer questions as limited due to other comorbidity

Individuals were asked whether they had been previously diagnosed with COPD, emphysema or chronic bronchitis alongside other common respiratory conditions. Data were collected around lung cancer risk factors including smoking history, education level, ethnicity, and personal and family history of lung cancer. Height and weight were measured to calculate body mass index (BMI) and spirometry reference values.

### Definition of COPD

For the purposes of this analysis, we defined clinically significant COPD as the presence of symptomatic airflow obstruction (FEV_1_/FVC ratio <0.7 on pre-bronchodilator spirometry) in association with positive symptoms (either cough of duration >6 weeks and/or dyspnoea with an mMRC score >1). This definition was adapted from the Global Initiative for Chronic Obstructive Lung Disease (GOLD) 2019 guidelines which advocated the consideration of COPD in those with dyspnoea, chronic cough or sputum production in those with exposures to the disease [29], and mirrored an approach used within other case-finding studies, including a pilot LCS population [9, 18, 30]. “Undiagnosed COPD” was defined as those who met the criteria for symptomatic airflow obstruction who did not self-report a previous diagnosis of COPD.

Subsequent analysis by clinical phenotypes characterised COPD by airflow limitation *via* GOLD grades 1–4 and separately by symptom/exacerbation burden through GOLD ABCD groupings (A, B, C or D) [22]. For the purposes of this analysis and based on the data-points collected as part of the LHC, we defined “chronic bronchitis” as the presence of cough productive of sputum for >12 months and “frequent exacerbator” as two or more exacerbations of at least moderate severity [31] in the past year.

### Management of undiagnosed COPD

Those who met the criteria for undiagnosed COPD were referred to primary care with a recommendation for consideration of diagnostic spirometry. Participants with asymptomatic airflow obstruction were invited back for re-assessment of symptoms and spirometry at their next annual visit. All current smokers were given very brief advice around smoking cessation and offered referral to a local smoking cessation service.

### LDCT and suspicious findings

LDCT scans were performed in the supine position at maximal inspiration. Scans were reported by trained thoracic radiologists into a bespoke template allowing categorisation of pulmonary nodules and incidental findings, including qualitative grading of the degree of emphysema by visual assessment as mild, moderate, severe or very severe. Findings suspicious for lung cancer were referred by the study team into the appropriate local lung multidisciplinary team (MDT) meeting. Detailed cancer outcome data were collected at 3 months following referral.

### Statistical analysis

Statistical analysis was undertaken using SPSS version 25 (IBM, Armonk, NY, USA). Four comparative analyses were undertaken to assess differences between 1) groups with and without airflow obstruction, 2) those with symptomatic and asymptomatic airflow obstruction, 3) those with undiagnosed and diagnosed symptomatic airflow obstruction, and 4) those deemed eligible for inclusion into LCS due to higher lung cancer risk and those deemed ineligible. Differences between groups were assessed using the two-sample independent t-test (parametric data) and Mann–Whitney U-test (nonparametric data) for continuous data variables. Statistical significance was defined through p-values <0.05. Differences between categorical data were assessed using Chi-squared tests, with additional analyses using the Bonferroni test to assess for differences between groups in variables with more than two categorical values.

Univariable and multivariable binary logistic regression analyses were used to test whether airflow obstruction was independently predictive of lung cancer risk on baseline LDCT. Multivariate models were adjusted for variables known to be predictive of lung cancer (age, sex, smoking status, pack-years, self-reported COPD, BMI, education level, ethnicity, personal history of cancer, family history of lung cancer and radiological emphysema). Additional multivariate analyses were performed to understand if the presence of symptoms and previous diagnosis were associated with increased lung cancer risk.

## Results

**FIGURE 1:**
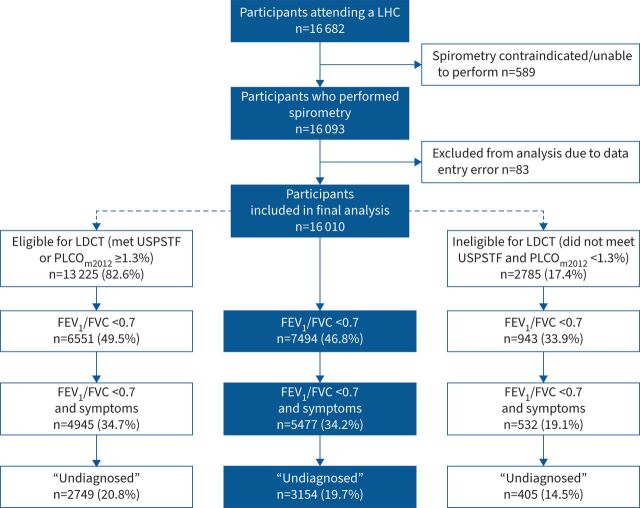
CONSORT (Consolidated Standards of Reporting Trials) diagram demonstrating all those attending a lung health check (LHC) between 8 April 2019 and 19 March 2020 and associated outcome following the SUMMIT targeted approach to case-finding for COPD. Rates of airflow obstruction, symptomatic airflow obstruction and undiagnosed symptomatic airflow obstruction are shown for the whole population (middle column). Additional subdivisions highlight these rates in those found to be eligible or ineligible for low-dose computed tomography (LDCT) lung cancer screening based on predicted lung cancer risk. USPSTF: US Preventive Services Task Force; PLCO_m2012_: Prostate, Lung, Colorectal and Ovarian Study Risk Prediction Model modified 2012; FEV_1_: forced expiratory volume in 1 s; FVC: forced vital capacity.

Figure 1 demonstrates the overall outcomes following the SUMMIT targeted approach to case-finding for undiagnosed COPD. 16 682 individuals attended a LHC from April 2019 to March 2020. Of these, 96.4% (16 093) attendees successfully performed spirometry. 16 010 individuals were included in the final analysis. 58.5% of attendees were male, with a mean age of 65.0 (95% CI 64.9–65.0) years and a mean pack-year history of 40.3 (95% CI 39.2–40.7) years. 60.8% left school at or before age 16 years and 61.4% lived in areas categorised within the two most deprived socioeconomic quintiles.

### Rates of COPD (symptomatic airflow obstruction)

46.8% (7494) of all attendees to a LHC demonstrated evidence of airflow obstruction on pre-bronchodilator spirometry. Symptomatic airflow obstruction (COPD) was demonstrated in 34.2% (n=5477) of attendees (73.1% of those with airflow obstruction). Table 2 details a comparison of the baseline characteristics of those with symptomatic airflow obstruction against those with asymptomatic airflow obstruction. The proportion of females was greater in those with symptomatic airflow obstruction (43.4% *versus* 35.5%; p<0.001) and this group were more frequently current smokers (51.8% *versus* 45.7%; p<0.001). Those with symptomatic airflow obstruction had a lower mean FEV_1_ % pred than those without symptoms (64.2% (95% CI 63.7–64.7%) *versus* 74.8% (95% CI 74.0–75.6%)).

**TABLE 2:** Baseline characteristics^#^ of the SUMMIT population with airflow obstruction

	**Airflow obstruction**			**Symptomatic airflow obstruction**		
	**Symptomatic (n=5477)**	**Asymptomatic (n=2017)**	**p-value**	**Undiagnosed (n=3154)**	**Diagnosed (n=2323)**	**p-value**
**Age (years)**	66.4 (66.2–66.5)	65.7 (65.4–65.9)	<0.001	66.1 (65.9–66.3)	66.7 (66.5–67.0)	<0.001
**Male**	3099 (56.6)	1301 (64.5)	<0.001	1863 (59.1)	1236 (53.2)	<0.001
**BMI (kg·m^−2^)**	27.8 (27.5–28.1)	26.4 (25.9–26.9)	<0.001	27.9 (27.5–28.3)	27.8 (27.2–28.3)	0.774
**Family history of lung cancer**	1001 (18.3)	285 (14.1)	<0.001	518 (16.4)	483 (20.8)	<0.001
**Personal history of cancer**	797 (14.6)	271 (13.4)	0.220	430 (13.6)	367 (15.8)	0.025
**Self-reported COPD**	2323 (42.4)	381 (18.9)	<0.001			
**Median (IQR) PLCO_m2012_ score (%)**	3.8 (1.9–7.1)	2.5 (1.3–4.5)	<0.001	2.9 (1.6–5.5)	5.3 (2.9–9.3)	<0.001
**Meeting PLCO_m2012_ ≥1.3%**	4749 (86.7)	1531 (75.9)	<0.001	2583 (81.9)	2166 (93.2)	<0.001
**Meeting USPSTF criteria**	4001 (73.1)	1185 (58.7)	<0.001	2227 (70.6)	1185 (58.7)	<0.001
**Pack-year history**	45.6 (44.9–46.3)	36.7 (35.7–37.6)	<0.001	43.4 (42.5–44.2)	48.6 (47.4–49.7)	<0.001
**Smoking status**			<0.001			<0.001
Current smoker	2839 (51.8)	922 (45.7)		1733 (54.9)	1106 (47.6)	
Former smoker	2637 (48.2)	1089 (54.0)		1420 (45.1)	1217 (52.4)	
Missing values	1 (0.0)	6 (0.3)		1 (0.0)	0 (0.0)	
**IMD quintile**			<0.001			
Quintile 1	1842 (33.6)	579 (28.7)		992 (31.5)	850 (36.6)	<0.001
Quintile 2	1654 (30.2)	533 (26.4)		921 (29.2)	733 (31.6)	
Quintile 3	928 (16.9)	396 (19.6)		574 (18.2)	354 (15.2)	
Quintile 4	755 (13.8)	347 (17.2)		466 (14.8)	289 (12.5)	
Quintile 5	239 (4.4)	139 (6.9)		161 (5.1)	78 (3.4)	
Missing values	59 (1.1)	23 (1.1)		40 (1.3)	19 (0.8)	
**Education level**			<0.001			<0.001
Finished school before age 15 years	2439 (44.5)	680 (33.7)		1314 (41.7)	1125 (48.4)	
Completed high school or equivalent	1259 (23.0)	428 (21.2)		721 (22.9)	538 (23.2)	
A-levels or equivalent	559 (2.9)	216 (10.7)		348 (11)	211 (9.1)	
Further education	408 (7.4)	212 (10.5)		264 (8.4)	144 (6.2)	
Bachelor degree	574 (10.5)	336 (16.7)		350 (11.1)	224 (9.6)	
Higher degree	238 (4.3)	145 (7.2)		157 (5.0)	81 (3.5)	
**Ethnicity**			<0.001			<0.001
Mixed	115 (2.1)	48 (2.4)		71 (2.3)	44 (1.9)	
Black	219 (4.0)	126 (6.2)		159 (5.0)	60 (2.6)	
Asian	402 (7.3)	125 (6.2)		291 (9.2)	111 (4.8)	
White	4568 (83.4)	1633 (81.0)		2506 (79.5)	2062 (88.8)	
Other	173 (3.2)	85 (4.2)		127 (4.0)	46 (2.0)	
**Spirometry**						
FEV_1_ (% pred)	64.2 (63.7–64.7)	74.8 (74.0–75.6)	<0.001	68.0 (67.4–68.6)	59.0 (58.2–59.7)	<0.001
**Symptoms**						
Cough >6 weeks				1039 (32.9)	926 (39.9)	<0.001
Sputum present				652 (20.7)	692 (29.8)	<0.001
mMRC score >1				2846 (90.2)	2201 (94.7)	<0.001
≥2 exacerbations in past year				138 (4.4)	516 (22.2)	<0.001

Data are presented as mean (95% confidence interval) or n (%), unless otherwise stated. BMI: body mass index; IQR: interquartile range; PLCO_m2012_: Prostate, Lung, Colorectal and Ovarian Study Risk Prediction Model modified 2012; USPSTF: US Preventive Services Task Force; IMD: Index of Multiple Deprivation; FEV_1_: forced expiratory volume in 1 s; mMRC: modified Medical Research Council. ^#^: baseline characteristics of 1) individuals with airflow obstruction (n=7494) stratified by the presence or absence of symptoms and 2) individuals with symptomatic airflow obstruction (n=5477) stratified by previous diagnosis.

### Rates of undiagnosed COPD

Undiagnosed symptomatic airflow obstruction was demonstrated in 19.7% (3154) of attendees (42% of all those with airflow obstruction). Table 2 shows a comparison of baseline characteristics between those with undiagnosed COPD and those with a previous diagnosis. Those undiagnosed had a higher mean FEV_1_ % pred and a lower overall symptom burden than those with a previous diagnosis. Those undiagnosed had a lower mean pack-year history (43.4 (95% CI 42.5–44.2) *versus* 48.6 (95% CI 47.4–49.7) pack-years; p<0.001), and were more likely to be male (59.1% *versus* 53.2%; p<0.001) and a current smoker (54.9% *versus* 47.6%; p<0.001). A greater proportion of those undiagnosed were from Black (5.0% *versus* 2.6%; p<0.001) and Asian (9.2% *versus* 4.8%; p<0.001) ethnic groups than those with diagnosed COPD. Those who were undiagnosed were more frequently from less deprived socioeconomic quintiles and whilst the majority of undiagnosed individuals had lower education levels, a slightly higher proportion had obtained an education level beyond high school equivalent than those diagnosed (35.1% *versus* 28.4%; p<0.001).

#### Clinical phenotypes

**FIGURE 2:**
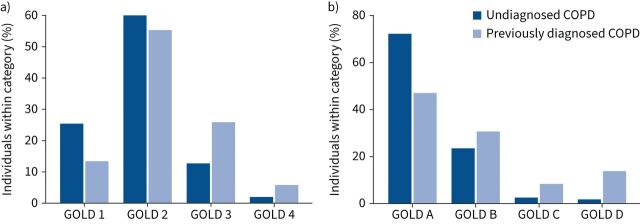
Prevalence and grade of a) airflow limitation and b) symptom and exacerbation burden between those with undiagnosed and previously diagnosed COPD. GOLD: Global Initiative for Obstructive Lung Disease.

In those with symptomatic airflow obstruction we compared clinical phenotypes between those with undiagnosed COPD and those with diagnosed COPD (table 2 and figure 2). Those undiagnosed were significantly more likely to be of a lower GOLD grade than those previously diagnosed (GOLD 1: 25.5% *versus* 13.3%; p<0.001; GOLD 2: 59.8% *versus* 55.2%; p<0.001; adjusted Bonferroni p=0.006). Similarly, when stratifying by GOLD ABCD group, those undiagnosed were significantly more likely to have lower symptom and exacerbation burden than those with a previous diagnosis (p<0.001). 72.0% of those undiagnosed were group A compared with 47% in those with a previous diagnosis (p<0.001; adjusted Bonferroni p=0.006) and only 1.9% of those undiagnosed fell into the highest symptom/exacerbation burden category of D compared with 13.9% of those previously diagnosed (p<0.001). Other common clinical phenotypes were significantly less frequent in those undiagnosed than those diagnosed. Those undiagnosed were less likely to have chronic bronchitis (13.5% *versus* 22.3%; p<0.001) or be a frequent exacerbator (4.4% *versus* 22.2%; p<0.001).

#### Undiagnosed COPD prevalence based on lung cancer risk

Of 16 010 attendees to a LHC, 13 225 (82.6%) participants were deemed eligible to undergo LDCT screening based on calculated lung cancer risk and 2785 (17.4%) were deemed ineligible. The median (interquartile range) 6-year PLCO_m2012_ score in those eligible was 3.1% (1.9–5.6%) compared with 0.7% (0.4–1.0%) in those deemed ineligible. Rates of airflow obstruction, symptomatic airflow obstruction and undiagnosed symptomatic airflow obstruction were all significantly lower in those ineligible for LDCT. Undiagnosed COPD was found in 14.5% (405) of those deemed ineligible for LCS compared with 20.8% (2749) of eligible individuals.

Of the 4790 individuals with undiagnosed airflow obstruction, 21.0% (1007) did not meet the PLCO_m2012_ eligibility threshold score of ≥1.3%. Substituting airflow obstruction into the PLCO_m2012_ model in this group would have resulted in an additional 339 individuals meeting the eligibility threshold. For the 3154 individuals with undiagnosed symptomatic airflow obstruction, 18.1% (n=571) did not meet a PLCO_m2012_ score of ≥1.3%. Substituting airflow obstruction into the PLCO_m2012_ model in this group would have resulted in an additional 242 individuals meeting the eligibility threshold.

### Cancer outcomes at 3 months post-baseline LDCT

11 115 out of 13 225 individuals eligible for LDCT on USPSTF or PLCO_m2012_ criteria underwent a baseline round of LDCT, with reasons for attrition loss including unwillingness to participate in research and inability to undergo LDCT for reasons such as claustrophobia. 382 (3.4%) participants had findings suspicious for lung cancer requiring referral into a local lung cancer MDT. Outcome cancer data at 3 months were available in 371 (97.1%) individuals and demonstrated 120 confirmed diagnoses of lung cancer. A further 122 individuals remained under surveillance by a lung MDT for suspicious lesions.

Lung cancer prevalence was significantly higher in those with airflow obstruction than those without (1.7% (91/5497) *versus* 0.5% (29/5618); p<0.001). On univariate binary logistic regression analysis, airflow obstruction was significantly associated with an increased risk of lung cancer (unadjusted OR 3.24, 95% CI 2.13–4.94; p<0.001). This association was independent of confounders adjusted for in multivariate analysis (adjusted OR 2.45, 95% CI 1.57–3.83; p<0.001) (table 3).

**TABLE 3:** Univariate and multivariate binary logistic regression analyses testing associations with the risk of lung cancer on baseline low-dose computed tomography at 3-month follow-up

	**Univariate**		**Multivariate**	
	**OR (95% CI)**	**p-value**	**OR (95% CI)**	**p-value**
**Age**				
Per increasing year	1.08 (1.05–1.11)	<0.001	1.05 (1.02–1.09)	0.002
**Sex**				
Male	1		1	
Female	1.50 (1.05–2.15)	0.028	1.39 (0.95–2.01)	0.089
**Pack-years**				
Per increasing pack-year	1.01 (1.00–1.01)	0.037	1.00 (1.00–1.01)	0.179
**Personal history of cancer**				
No	1			
Yes	2.11 (1.38–3.21)	<0.001	1.77 (1.15–2.72)	0.009
**Family history of lung cancer**				
No	1		1	
Yes	2.08 (1.41–3.05)	<0.001	2.02 (1.36–2.99)	<0.001
**Emphysema presence?**				
No	1		1	
Yes	2.01 (1.40–2.88)	<0.001	1.43 (0.97–2.10)	0.069
**Airflow obstruction?**				
No	1		1	
Yes	3.24 (2.13–4.94)	<0.001	2.45 (1.57–3.83)	<0.001
**Symptomatic airflow obstruction?**				
No airflow obstruction	1		1	
Asymptomatic	1.94 (1.00–3.73)	0.049	1.61 (0.82–3.16)	0.163
Symptomatic	3.66 (2.38–5.61)	<0.001	2.74 (1.73–4.34)	<0.001
**Previous diagnosis?**				
No airflow obstruction	1		1	
Asymptomatic	1.94 (1.00–3.73)	0.049	1.61 (0.82–3.16)	0.163
Undiagnosed symptomatic airflow obstruction	3.83 (2.40–6.13)	<0.001	2.79 (1.67–4.64)	<0.001
Diagnosed symptomatic airflow obstruction	3.44 (2.08–5.68)	<0.001	2.61 (1.15–5.91)	0.022

Multivariate models were adjusted for variables known to be predictive of lung cancer (age, sex, smoking status, pack-years, self-reported COPD, body mass index, education level, ethnicity, personal history of cancer, family history of lung cancer and radiological emphysema). A threshold of p<0.05 was considered significant.

Further inclusion of categories of airflow obstruction into the models found that symptomatic airflow obstruction remained a strong independent risk factor for lung cancer (adjusted OR 2.74, 95% CI 1.73–4.34; p<0.001). For those with symptomatic airflow obstruction, an independent increased association with lung cancer risk remained in both those with a previous diagnosis and those undiagnosed. However, the odds ratio was slightly higher in those with undiagnosed COPD (adjusted OR 2.79, 95% CI 1.67–4.60; p<0.001) than those with previously diagnosed COPD (adjusted OR 2.61, 95% CI 1.15–5.91; p=0.022).

Interestingly, whilst univariate analysis did show increased risk of lung cancer with emphysema, this did not remain significant on multivariate analysis (adjusted OR 1.43, 95% CI 0.97–2.10; p=0.069). Further insertion of categories of emphysema severity into the multivariate model still did not demonstrate any independent relationship with the degree of emphysema and lung cancer diagnosis.

## Discussion

In this large study of 16 010 individuals, we investigated the role of a targeted approach combining spirometry with respiratory symptom assessment to identify undiagnosed COPD in a LCS cohort. High rates of pre-bronchodilator airflow obstruction were found in nearly half (46.8%) of all attendees. Nearly one in five (19.7%) attendees had undiagnosed symptomatic COPD. Airflow obstruction was found to be an independent risk factor for lung cancer on baseline LDCT, with a high risk demonstrated in those with undiagnosed COPD (adjusted OR 2.79, 95% CI 1.67–4.64; p<0.001).

Rates of airflow obstruction in this study (46.8%) were significantly higher than those previously reported in the NLST-ACRIN cohort (34.5%) [32]. Our cohort was older, had a lower level of education and was more ethnically diverse than the NLST-ACRIN cohort, suggesting a more “real-world” population. Whilst airflow obstruction rates were slightly lower than those reported in the London-based Lung Screen Uptake Trial (LSUT) pilot study (56%), a much higher proportion of LSUT attendees were current smokers (70.9% *versus* 47.5%), reflecting the narrower criteria that study utilised for invitation to LCS [33]. We acknowledge that a significant limitation of our results is that our rates of airflow obstruction are based on pre-bronchodilator spirometry and therefore may overestimate the prevalence of undiagnosed COPD. Studies examining the difference between pre-bronchodilator and post-bronchodilator spirometry have demonstrated a reduction in COPD prevalence by 25–35% after bronchodilation [34–38]. However, our methodology of using pre-bronchodilator spirometry for the purposes of case-finding rather than diagnosis is consistent with previous studies assessing the utility of handheld spirometry in primary care [39–42] and that utilised by other LCS studies [13–15], and a recommendation was made to primary care to consider diagnostic spirometry in those found to meet our criteria of undiagnosed symptomatic COPD. Importantly, we employed a quality assurance policy to spirometry performance to maximise the accuracy of results obtained. We have demonstrated that this pragmatic approach can be performed on a large scale whilst maintaining a minimally invasive and time-sensitive approach to spirometry required in the context of LCS.

There are limited previous LCS studies that categorise undiagnosed COPD through the definition of undiagnosed symptomatic airflow obstruction rather than undiagnosed airflow obstruction [17, 18]. Our approach referred only those with undiagnosed symptomatic airflow obstruction to primary care for consideration of diagnostic spirometry. Consented participants with asymptomatic, undiagnosed airflow obstruction will be re-assessed at their next annual study visit. This approach reduced the immediate referral rate to primary care by 10% (29.9% with undiagnosed airflow obstruction *versus* 19.7% with undiagnosed symptomatic airflow obstruction), although we do acknowledge that additional referrals may be required if an asymptomatic individual becomes symptomatic at an onwards screening visit. However, this approach may best optimise the utilisation of health resources required as a result of case-finding and minimise harm through investigating asymptomatic individuals in whom current guidelines suggest no benefit.

Individuals in our study were more likely to have undiagnosed COPD if they were male, were a current smoker and had less overall tobacco exposure. Additionally, we found a higher prevalence of undiagnosed COPD in those from Black, Asian, Mixed and Other ethnic groups compared with those of a White ethnic background. These data are in keeping with previous studies that have identified being male, a current smoker and from an ethnic minority group as consistent risk factors for underdiagnosis of COPD [8, 9, 43]. Whilst the reasons for these disparities are not fully understood, patient healthcare beliefs alongside utilisation and access to healthcare services are thought to play a role in underdiagnosis in these groups. Our finding that those underdiagnosed had slightly higher education levels than those diagnosed is somewhat surprising, although the existing literature is conflicting as to whether education levels play a role in COPD underdiagnosis [8, 9]. However, the overall findings from our data suggest that incorporating case-finding for COPD into LCS programmes may be one effective method to help target those groups at the highest risk of COPD and in doing so reduce health inequalities.

The overall goal of case-finding for COPD is to reduce disease-related morbidity and mortality through timely interventions to reduce symptoms and risk of associated complications, notably exacerbations. Increasing recognition that FEV_1_ weakly correlates with symptom burden and quality of life in those with COPD has led to a greater importance being placed on symptom assessment to guide treatment [22]. To the best of our knowledge, we are the first LCS study to analyse prevalence of undiagnosed COPD by established clinical phenotypes other than FEV_1_ alone. Whilst those with undiagnosed COPD were more likely to be of a lower GOLD grade, indicating more preserved lung function, they were also more likely to be of a lower GOLD ABCD group and less likely to fit the “chronic bronchitis” or “frequent exacerbator” phenotypes. Lower overall symptom burden is known to be associated with underdiagnosis of COPD as individuals may be less likely to seek medical attention and healthcare practitioners less likely to suspect a diagnosis of COPD until symptoms are more advanced [8, 9]. This suggests that there may be increased benefit in utilising case-finding for COPD within LCS programmes to induce a “stage shift” in earlier COPD diagnosis, potentially allowing intervention before significant impingement on quality of life and functional reserve. The clinical benefits of such an approach may maximise the value of LCS beyond lung cancer detection alone.

The concept of using a LHC to determine eligibility into LCS is relatively new, with a desired aim of increasing the clinical benefits beyond lung cancer detection alone. To the best of our knowledge, this is the first study to compare the yield from case-finding as part of a LHC between those eligible and those ineligible for inclusion into LCS. The prevalence of undiagnosed COPD was expectedly lower in those deemed ineligible to take part in LCS based on predicted lung cancer risk. This is likely explained by the fact that this group were younger with less tobacco exposure. However, rates of undiagnosed COPD were still high in those found to be ineligible for LDCT screening, with 14.5% having undiagnosed COPD. This finding adds a new layer of evidence to suggest the potential clinical benefits of the LHC approach even in those found to be ineligible for LCS.

Limited previous data have suggested that the use of spirometry may increase the accuracy of lung cancer risk prediction models [44, 45]. As the largest prospective dataset in a LCS population, our data add further weight to this argument. Using preliminary cancer outcome data, we have demonstrated that the presence of airflow obstruction independently increases the risk of lung cancer on a baseline round of LDCT by 2.45 times. Furthermore, when comparing those with previously diagnosed and undiagnosed disease, the lung cancer risk persisted in those with undiagnosed COPD (adjusted OR 2.79, 95% CI 1.67–4.6; p<0.001). Given the high rates of undiagnosed COPD in our population, this suggests that risk prediction models that rely on self-reported COPD may underestimate lung cancer risk in this group, denying them the opportunity to undergo screening. An extra 339 individuals with undiagnosed airflow obstruction (2.5% of the total population attending a LHC) would have met the PLCO_m2012_ eligibility threshold had spirometry been utilised rather than self-reported COPD. Interestingly, our data do suggest that lung cancer risk is highest in those with symptoms and airflow obstruction. However, we acknowledge that our cancer outcomes are limited by a short period of follow-up and this will particularly underestimate stage 1 cancers requiring longer term follow-up; therefore, we cannot draw definitive conclusions from this preliminary analysis. Our data are further limited by a lack of cancer outcome data in those found to be ineligible. Through the use of national registry data, longer term cancer outcomes on those deemed eligible and ineligible for LDCT screening within the SUMMIT Study will be ascertained to understand the risk of lung cancer in the presence of airflow obstruction more accurately. Additionally, we acknowledge that whilst not the primary focus of this analysis, we found no association between the presence of emphysema and lung cancer risk. Whether it is airflow obstruction or emphysema that is the primary driver of lung cancer risk has been investigated within several studies but the results have been contradictory [4, 5, 46–52]. Further work assessing lung cancer risk in our population in association with longer term cancer outcome data is required to understand these associations more fully.

### Conclusions

This study demonstrates a high prevalence of undiagnosed clinically significant airflow obstruction in a LCS cohort. We demonstrate that a targeted approach combining spirometry and respiratory symptom assessment can facilitate case-finding for COPD on a large scale. Our results suggest that incorporating targeted case-finding into LCS programmes may be an opportunity to reach those at the highest risk of COPD underdiagnosis and facilitate a stage shift in earlier COPD diagnosis. Preliminary data suggest that those with undiagnosed COPD are at high risk of lung cancer and may gain benefit from inclusion into screening programmes. Longer term data on COPD and lung cancer outcomes will be utilised to understand the clinical utility of our approach and understand whether the incorporation of spirometry into LCS eligibility assessment increases the accuracy of lung cancer risk prediction.

## Supplementary material

10.1183/13993003.00795-2022.Supp1 **Please note:** supplementary material is not edited by the Editorial Office, and is uploaded as it has been supplied by the author.
Supplementary material: the SUMMIT Consortium ERJ-00795-2022.Supplement

## Shareable PDF

10.1183/13993003.00795-2022.Shareable1 This one-page PDF can be shared freely online.
Shareable PDF ERJ-00795-2022.Shareable
