# Supplementary material for: Detection of COPD in the SUMMIT Study lung cancer screening cohort using symptoms and spirometry
Source: Eur Respir J. 2022 Dec 8;60(6):2200795. doi: 10.1183/13993003.00795-2022 (PMC10436757; doi:10.1183/13993003.00795-2022)
Supplement: Supplementary file 1 [file ERJ-00795-2022.Supplement.pdf]

The SUMMIT Consortium

Sam M Janes[1], Jennifer L Dickson[1], Carolyn Horst[1], Sophie Tisi[1], Helen Hall[1], Priyam Verghese[1], Andrew Creamer[1], Thomas Callender[1], Ruth Prendecki[1], Amyn Bhamani[1], Mamta Ruparel[1], Allan Hackshaw[2], Laura Farrelly[2], Jon Teague[2], Anne-Marie Mullin[2], Kitty Chan[2], Rachael Sarpong[2], Malavika Suresh[2], Samantha L Quaife[3], Arjun Nair[4] , Anand Devaraj[5] ,[6] , Kylie Gyertson[4] , Vicky Bowyer[4] , Ethaar El-Emir [4] , Judy Airebamen[4] , Alice Cotton[4] , Kaylene Phua[4] , Elodie Murali[4] , Simranjit Mehta[4] , Janine Zylstra[4] , Karen Parry-Billings[4] , Columbus Ife[4] , April Neville[4] , Paul Robinson[4] , Laura Green[4] , Zahra Hanif[4] , Helen Kiconco[4] , Ricardo McEwen[4] , Dominique Arancon[4] , Nicholas Beech[4] , Derya Ovayolu[4] , Christine Hosein[4] , Sylvia Patricia Enes[4] , Qin April Neville[4] , Jane Rowlands[4] , Aashna Samson[4] , Urja Patel[4] , Fahmida Hoque[4] , Hina Pervez[4] , Sofia Nnorom[4] , Moksud Miah[4] , Julian McKee[4] , Mark Clark[4] , Jeannie Eng[4] , Fanta Bojang[4] , Claire Levermore[4] , Anant Patel[7] , Sara Lock[8] , Rajesh Banka[9] , Angshu Bhowmik[10] , Ugo Ekeowa[11], Zaheer Mangera[12], William M Ricketts[13], Neal Navani[4] , Terry O'Shaughnessy[13], Charlotte Cash[7] , Magali Taylor[4] , Samanjit Hare[7] , Tunku Aziz[13], Stephen Ellis[13], Anthony Edey[14] , Graham Robinson[15] , Alberto Villanueva[16] , Hasti Robbie[17] , Elena Stefan[18] , Charlie Sayer[19] , Nick Screaton[20] , Navinah Nundlall[4] , Lyndsey Gallagher[4] , Andrew Crossingham[4] , Thea Buchan[4] , Tanita Limani[4] , Kate Gowers[1], Kate Davies[1], John McCabe[1], Joseph Jacob[1],[24] , Karen Sennett[21], Tania Anastasiadis [22], Andrew Perugia[23], James Rusius[23].

1 Lungs For Living Research Centre, UCL Respiratory, University College London, London
2 CRUK & UCL Cancer Trials Centre, University College London, London
3 Centre for Prevention, Detection and Diagnosis, Wolfson Institute of Population Health, Barts and The London School of Medicine and Dentistry, Queen Mary University of London, London
4 University College London Hospitals NHS Foundation Trust, London
5 Royal Brompton and Harefield NHS Foundation Trust, London
6 National Heart and Lung Institute, Imperial College, London
7 Royal Free London NHS Foundation Trust, London
8 Whittington Health NHS Trust, London
9 Barking, Havering and Redbridge University Hospitals NHS Trust, Essex
10 Homerton University Hospital Foundation Trust, London
11 The Princess Alexandra Hospital NHS Trust, Essex
12 North Middlesex University Hospital NHS Trust, London
13 Barts Health NHS Trust, London
14 North Bristol NHS Trust, Bristol
15 Royal United Hospitals Bath NHS Foundation Trust, Bath
16 Surrey and Sussex Healthcare NHS Trust, Surrey
17 King's College Hospital NHS Foundation Trust, London
18 The Princess Alexandra Hospital NHS Trust, London
19 University Hospitals Sussex NHS Foundation Trust, Sussex
20 Royal Papworth Hospital NHS Foundation Trust, Cambridge
21 Killick Street Health Centre, London
22 Tower Hamlets Clinical Commissioning Group, London
23 Noclor Research Support, London
24 Centre for Medical Image Computing (CMIC), London
