# Supplementary material for: Detection of COPD in the SUMMIT Study lung cancer screening cohort using symptoms and spirometry
Source: Eur Respir J. 2022 Dec 8;60(6):2200795. doi: 10.1183/13993003.00795-2022 (PMC10436757; doi:10.1183/13993003.00795-2022)
Supplement: Supplementary file 2 [file ERJ-00795-2022.Shareable.pdf]

EUROPEAN RESPIRATORY JOURNAL
ORIGINAL RESEARCH ARTICLE
S. TISI ET AL.

Detection of COPD in the SUMMIT Study lung cancer screening cohort using symptoms and spirometry

Sophie Tisi[1], Jennifer L. Dickson[1], Carolyn Horst[1], Samantha L. Quaife[2], Helen Hall[1], Priyam Verghese[1], Kylie Gyertson[3], Vicky Bowyer[3], Claire Levermore[3], Anne-Marie Mullin[4], Jonathan Teague[4], Laura Farrelly[4], Arjun Nair[3], Anand Devaraj[5], Allan Hackshaw[4], the SUMMIT Consortium[6], John R. Hurst[1] and Sam M. Janes[1]

[1]Lungs for Living Research Centre, UCL Respiratory, University College London, London, UK. [2]Centre for Prevention, Detection and Diagnosis, Wolfson Institute of Population Health, Barts and The London School of Medicine and Dentistry, Queen Mary University of London, London, UK. [3]University College London Hospitals NHS Foundation Trust, London, UK. [4]Cancer Research UK and UCL Cancer Trials Centre, University College London, London, UK. [5]Royal Brompton and Harefield NHS Foundation Trust, London, UK. [6]Full details of the SUMMIT Consortium and affiliations are included in the supplementary material.

Corresponding author: Sam M. Janes (s.janes@ucl.ac.uk)

Shareable abstract (@ERSpublications)
Targeted case-finding using symptom assessment and spirometry within a large-scale lung cancer screening setting detects high rates of early-stage, clinically significant undiagnosed COPD. Those with undiagnosed COPD are at high risk for lung cancer https://bit.ly/3bo5piE

Cite this article as: Tisi S, Dickson JL, Horst C, *et al*. Detection of COPD in the SUMMIT Study lung cancer screening cohort using symptoms and spirometry. *Eur Respir J* 2022; 60: 2200795 [DOI: 10.1183/13993003.00795-2022].

This single-page version can be shared freely online.

Copyright ©The authors 2022.

This version is distributed under the terms of the Creative Commons Attribution Licence 4.0.

This article has an editorial commentary: https://doi.org/10.1183/13993003.01294-2022

Received: 20 April 2022
Accepted: 13 June 2022

Abstract

Background COPD is a major comorbidity in lung cancer screening (LCS) cohorts, with a high prevalence of undiagnosed COPD. Combining symptom assessment with spirometry in this setting may enable earlier diagnosis of clinically significant COPD and facilitate increased understanding of lung cancer risk in COPD. In this study, we wished to understand the prevalence, severity, clinical phenotype and lung cancer risk of individuals with symptomatic undiagnosed COPD in a LCS cohort.

Methods 16 010 current or former smokers aged 55–77 years attended a lung health check as part of the SUMMIT Study. A respiratory consultation and spirometry were performed alongside LCS eligibility assessment. Those with symptoms, no previous COPD diagnosis and airflow obstruction were labelled as undiagnosed COPD. Baseline low-dose computed tomography (LDCT) was performed in those at high risk of lung cancer ($PLCO_{m2012}$ score ⩾1.3% and/or meeting USPSTF 2013 criteria).

Results Nearly one in five (19.7%) met criteria for undiagnosed COPD. Compared with those previously diagnosed, those undiagnosed were more likely to be male (59.1% *versus* 53.2%; p<0.001), currently smoking (54.9% *versus* 47.6%; p<0.001) and from an ethnic minority group (p<0.001). Undiagnosed COPD was associated with less forced expiratory volume in 1 s impairment (Global Initiative for Chronic Obstructive Lung Disease (GOLD) grades 1 and 2: 85.3% *versus* 68.4%; p<0.001) and lower symptom/exacerbation burden (GOLD A and B groups: 95.6% *versus* 77.9%; p<0.001) than those with known COPD. Multivariate analysis demonstrated that airflow obstruction was an independent risk factor for lung cancer risk on baseline LDCT (adjusted OR 2.74, 95% CI 1.73–4.34; p<0.001), with a high risk seen in those with undiagnosed COPD (adjusted OR 2.79, 95% CI 1.67–4.64; p<0.001).

Conclusions Targeted case-finding within LCS detects high rates of undiagnosed symptomatic COPD in those most at risk. Individuals with undiagnosed COPD are at high risk for lung cancer.
